# Supplementary material for: Wafer-scale organic-on-III-V monolithic heterogeneous integration for active-matrix micro-LED displays
Source: Nat Commun. 2023 Nov 1;14:6985. doi: 10.1038/s41467-023-42443-8 (PMC10620182; doi:10.1038/s41467-023-42443-8)
Supplement: Supplementary file 1 — Supplementary Information [file 41467_2023_42443_MOESM1_ESM.pdf]

Wafer-scale Organic-on-III-V Monolithic Heterogeneous Integration for Active-Matrix Micro-LED Displays

Lei Han[1,2], Simon Ogier[3], Jun Li[1,2], Dan Sharkey[3], Xiaokuan Yin[1,2], Andrew Baker[3], Alejandro Carreras[3], Fangyuan Chang[4], Kai Cheng[5], Xiaojun Guo[1,2]*

[1]School of Electronic Information and Electrical Engineering, Shanghai Jiao Tong University, Shanghai, 200240, P.R. China.

[2]National Key Laboratory of Science and Technology on Micro/Nano Fabrication, Shanghai Jiao Tong university, Shanghai, 200240, P.R. China

[3]SmartKem Ltd., Neville Hamlin Building, Thomas Wright Way, NetPark, Sedgefield, TS21 3FG, UK.

[4]School of Design, Shanghai Jiao Tong University, Shanghai, 200240, P.R. China.

[5]Enkris Semiconductor, Inc., Nanopolis Suzhou, 99 Jinji Avenue, Suzhou Industrial Park, Suzhou, Jiangsu Province, 215124, P.R. China.

*Corresponding author, Email: x.guo@sjtu.edu.cn

Section 1 Supplementary Figures

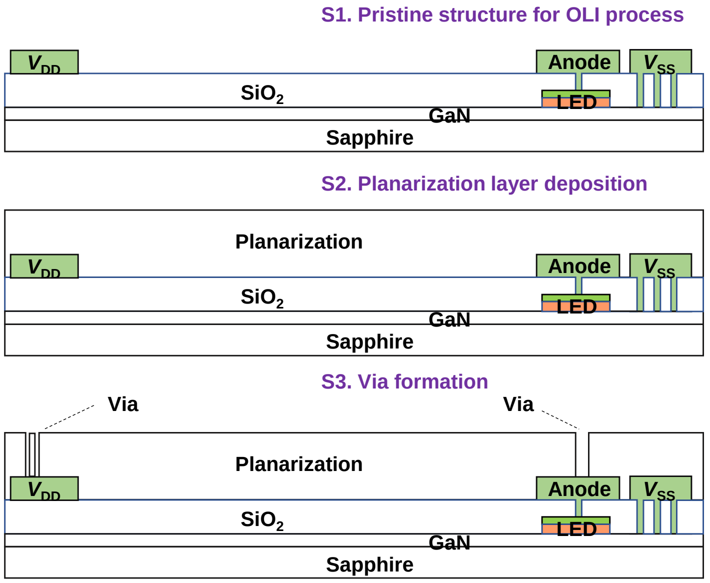

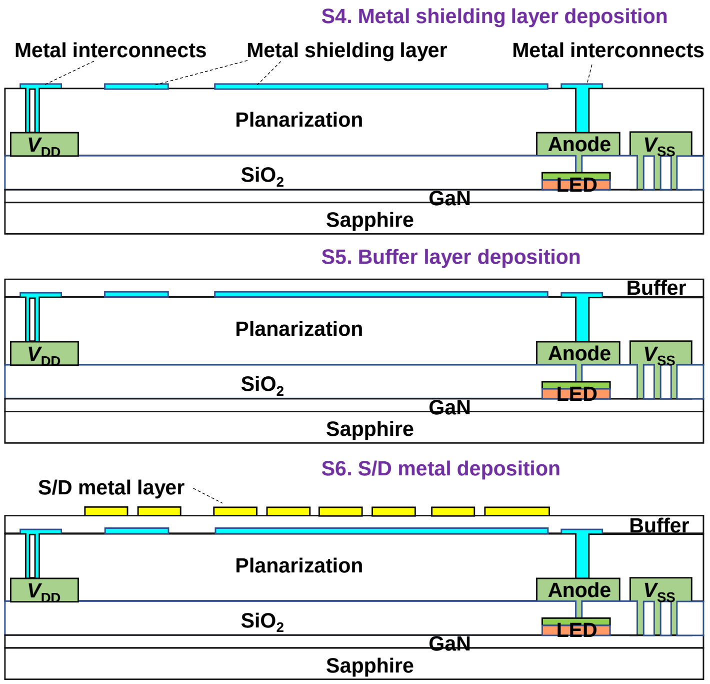

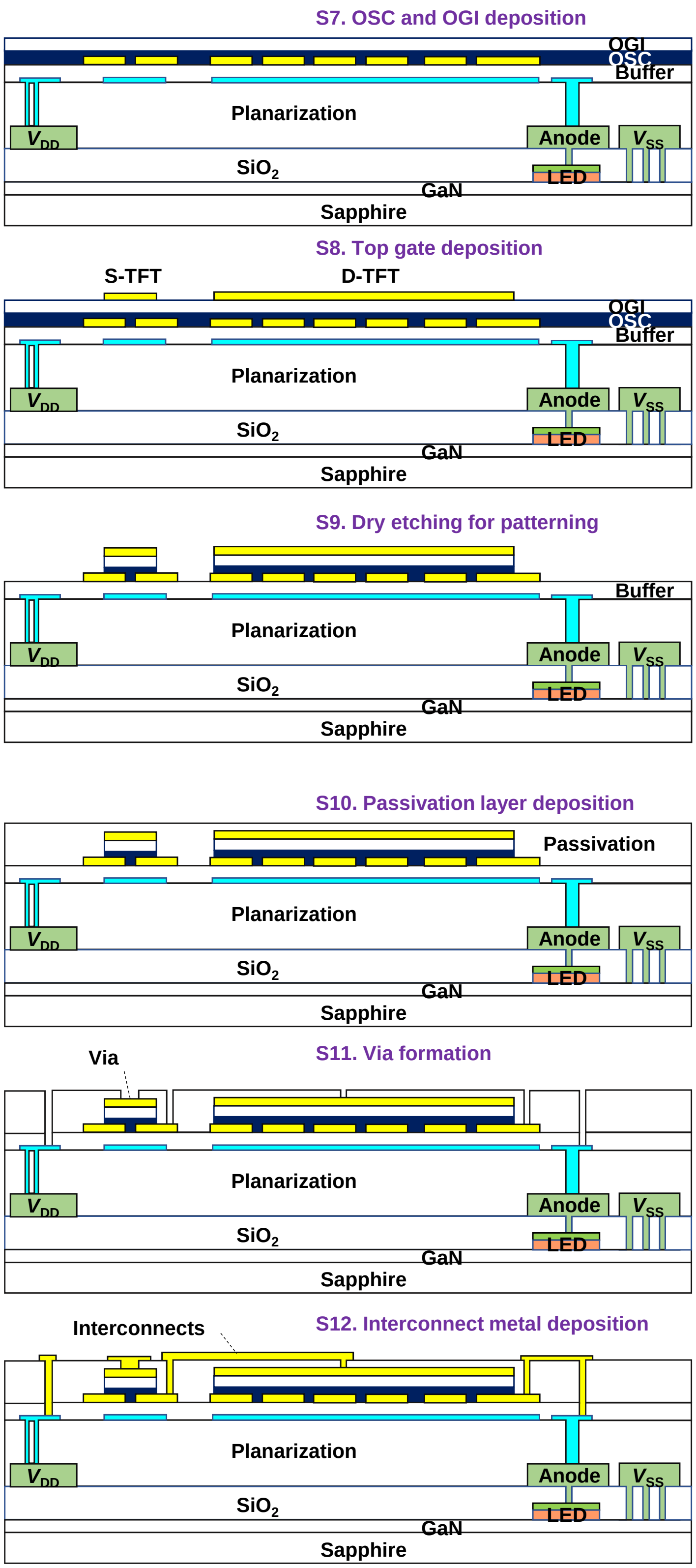

Supplementary Fig. 1 Process flow of organic last integration (OLI) directly onto micro-LED wafer.

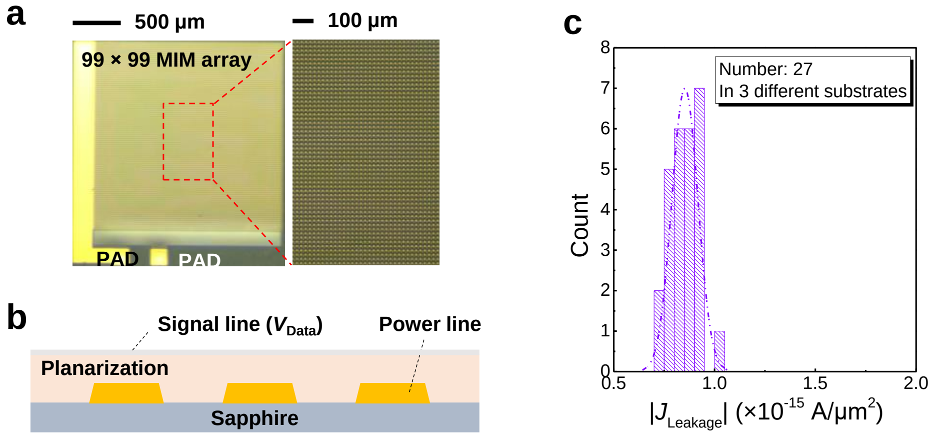

Supplementary Fig. 2 Leakage property of planarization layer in enhancing electrical reliability: (a) Optical image of metal-insulator-metal (MIM) array in parallel connection. (b) Schematic of cross-sectional structure of the MIM array. (c) Statistic distribution of leakage current density of planarization layer based on 27 MIM devices locating uniformly in 3 different samples.

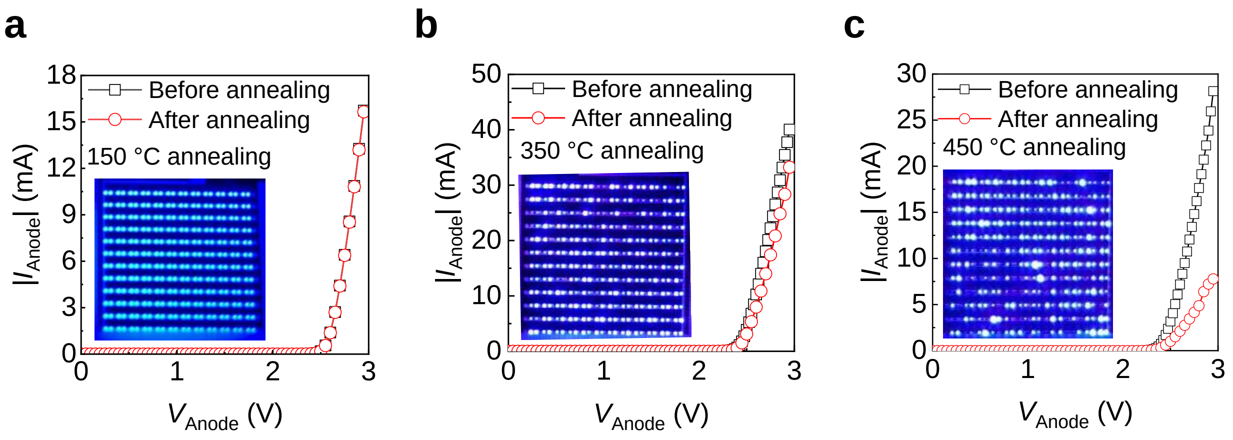

Supplementary Fig. 3 Characterization of the micro-LED performance before and after annealing under different temperature: (a) 150 ℃. (b) 350 ℃. (c) 450 ℃.

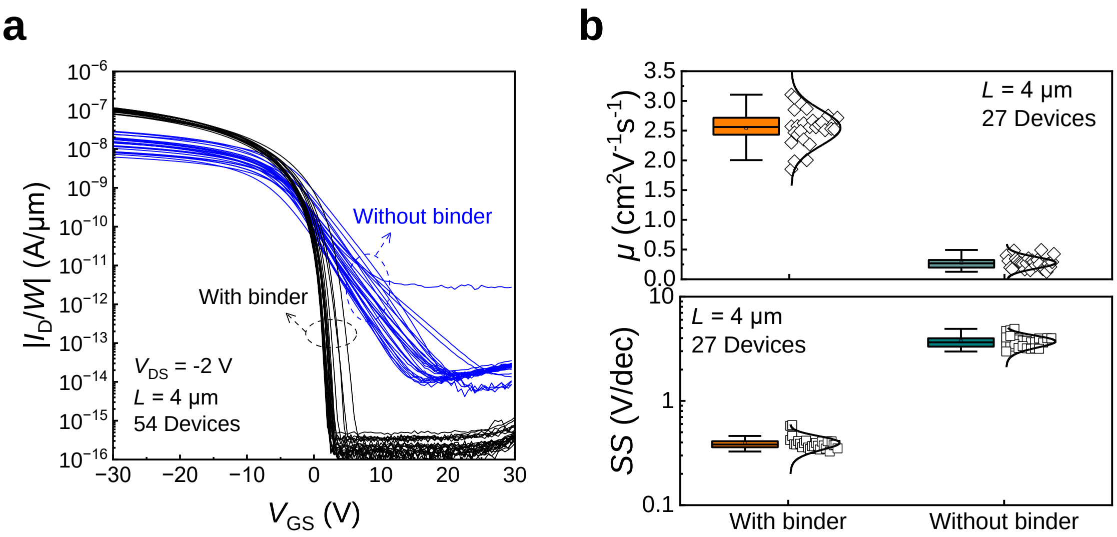

Supplementary Fig. 4 Performance comparison of OTFTs with/without introducing polymer semiconductor binder: (a) Measured transfer characteristics ($|I_D|$-V_{GS}) of 54 different devices. (b) Statistic distribution of mobility (μ) and subthreshold swing (SS).

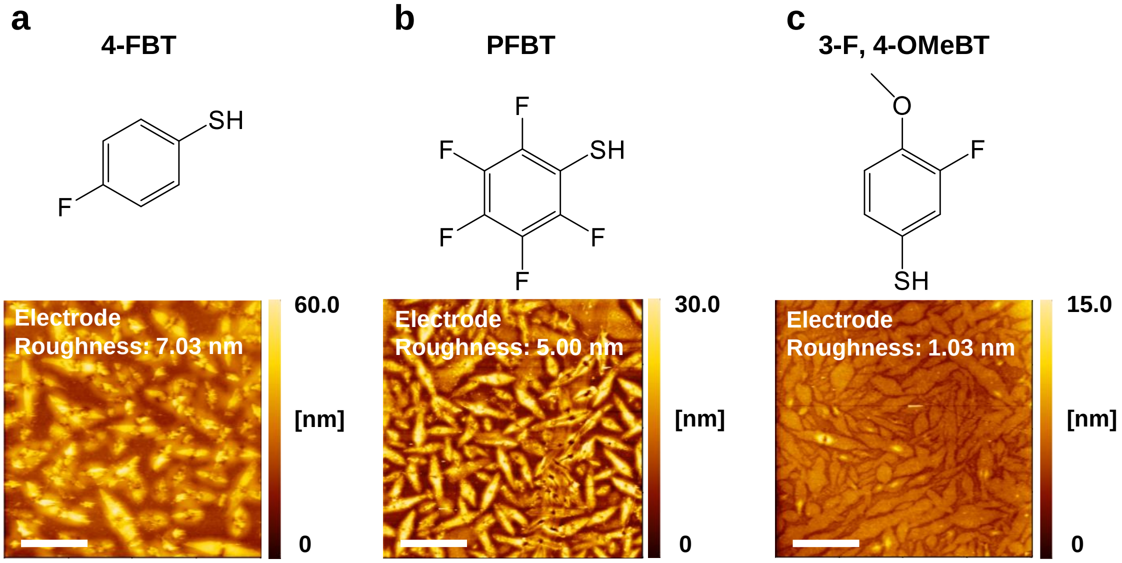

Supplementary Fig. 5 Surface roughness of OSC onto electrodes with different SAMs modification: (a-c) Molecule structures of different SAMs (4-Fluorothiophenol, 4-FBT; Pentafluorothiophenol, PFBT; 3-Fluoro-4-Methoxythiophenol, 3-F, 4-OMeBT) and

corresponding atomic force micrographs (AFM) of OSC onto electrodes with different SAMs modification. Scale bar: 10 μm.

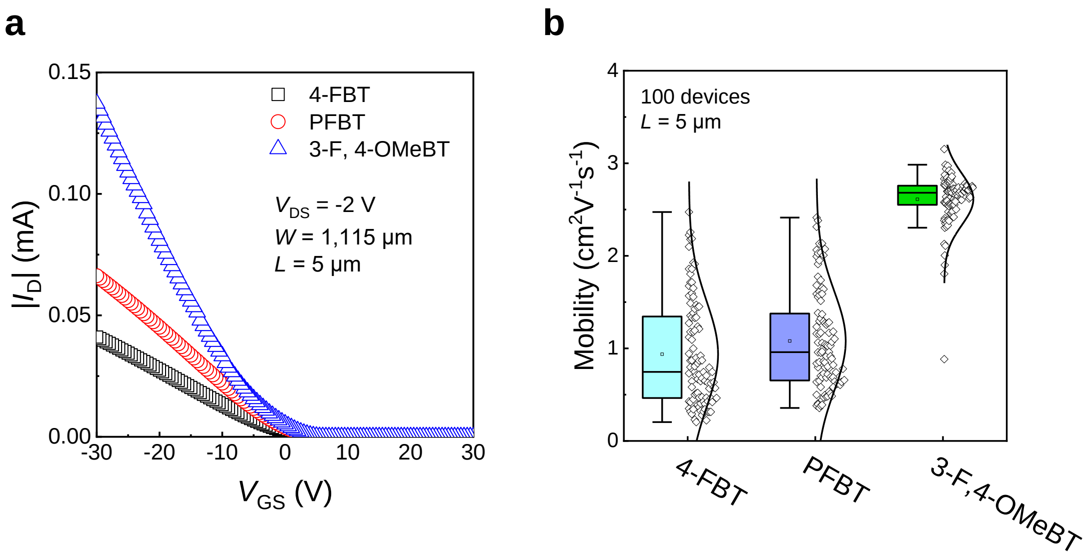

Supplementary Fig. 6 Electrical performance of devices with different self-assembled monolayers (SAMs) modification (4-Fluorothiophenol, 4-FBT; Pentafluorothiophenol, PFBT; 3-Fluoro-4-Methoxythiophenol, 3-F, 4-OMeBT): (a) Measured transfer characteristics ($|I_D|$-V_{GS}) and (b) statistic distribution of mobility (μ).

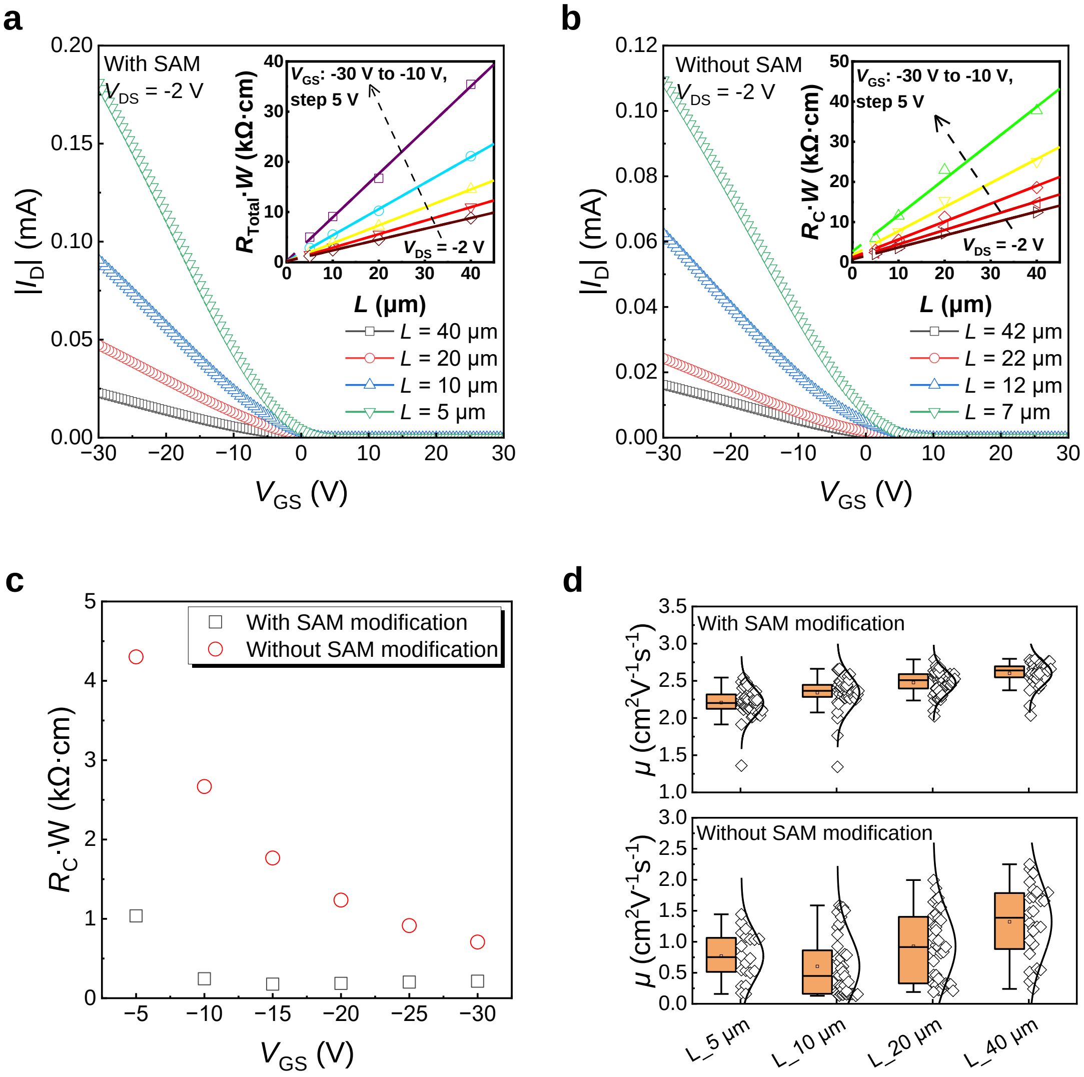

Supplementary Fig. 7 Electrical performance of OTFT with/without self-assembled monolayer (SAM) modification: (a, b) Measured transfer characteristics ($|I_D|$-V_{GS}) and extracted contact resistance of devices. (c) Evolution of contact resistance under different V_{GS}. (d) Comparison of mobility (μ) for devices with different channel length.

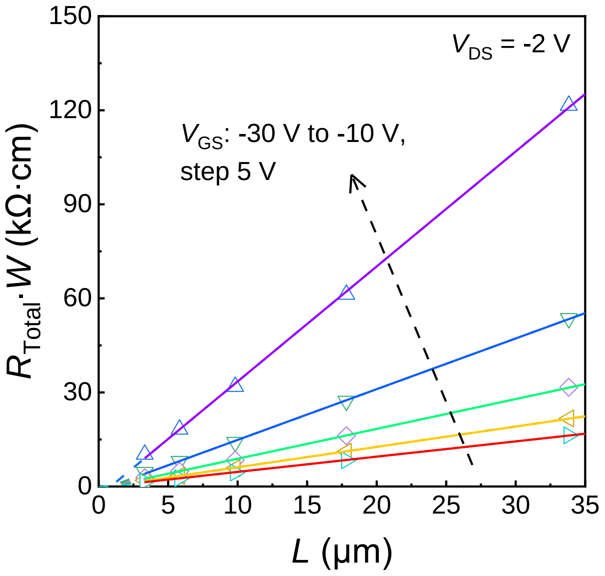

Supplementary Fig. 8 Extracted contact resistance (R_C) of device using transmission line measurement (TLM) method.

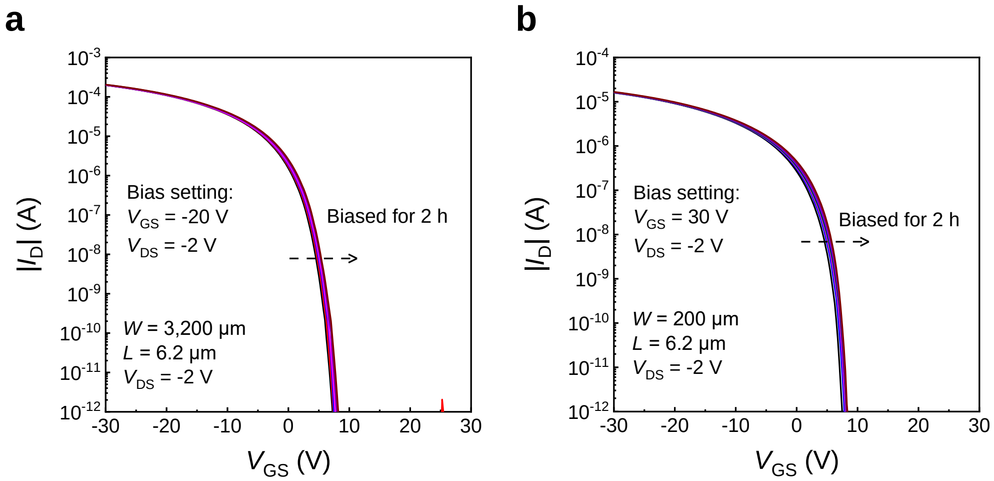

Supplementary Fig. 9 Electrical bias stress stability of device in ambient atmosphere: (a) Negative bias stress (NBS) stability. (b) Positive bias stress (PBS) stability.

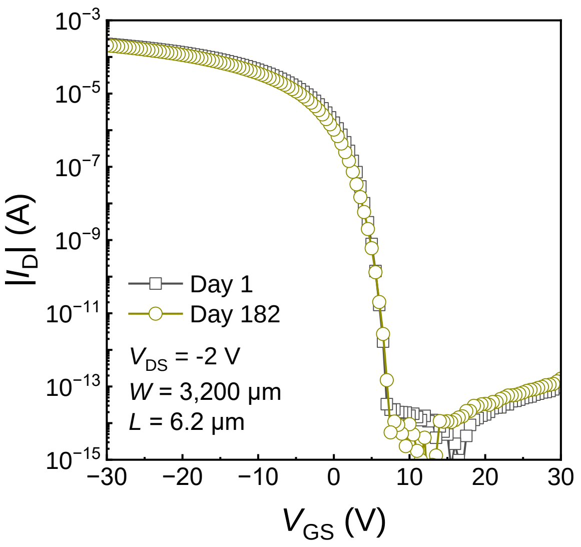

Supplementary Fig. 10 Measured transfer characteristics (|I_D|-V_{GS}) of the same device after 182 days storage by applying a short time annealing (80 °C for 10 minutes).

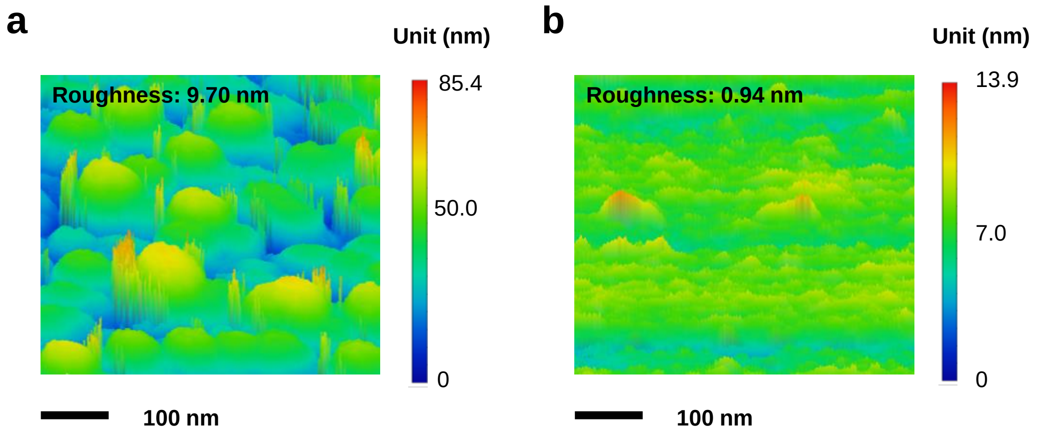

Supplementary Fig. 11 Atomic force micrographs (AFM) of buffer layers: (a) after being exposed to 20 minutes O_2 plasma. (b) without being exposed to O_2 plasma.

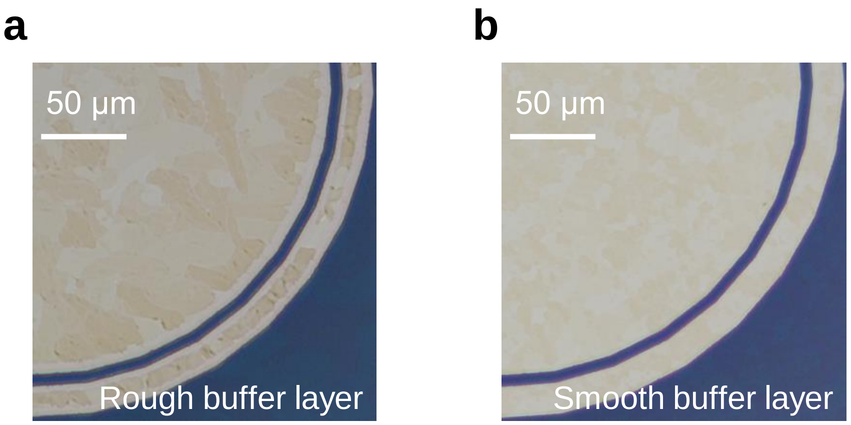

Supplementary Fig. 12 Polarized optical micrograph (POM) of OSC onto buffer layers with different roughness: (a) Rough buffer layer. (b) Smooth buffer layer.

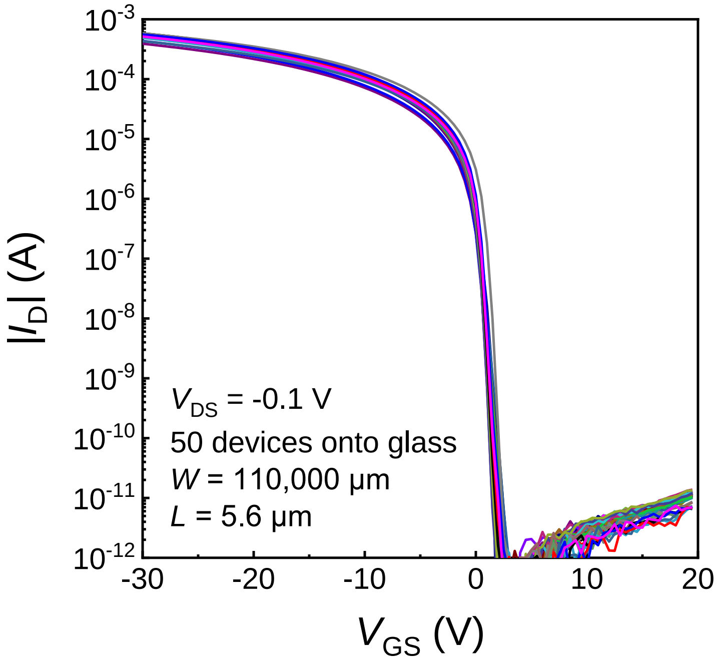

Supplementary Fig. 13 Measured transfer characteristics ($|I_D|$-V_{GS}) of 50 different devices based on glass substrate for reference.

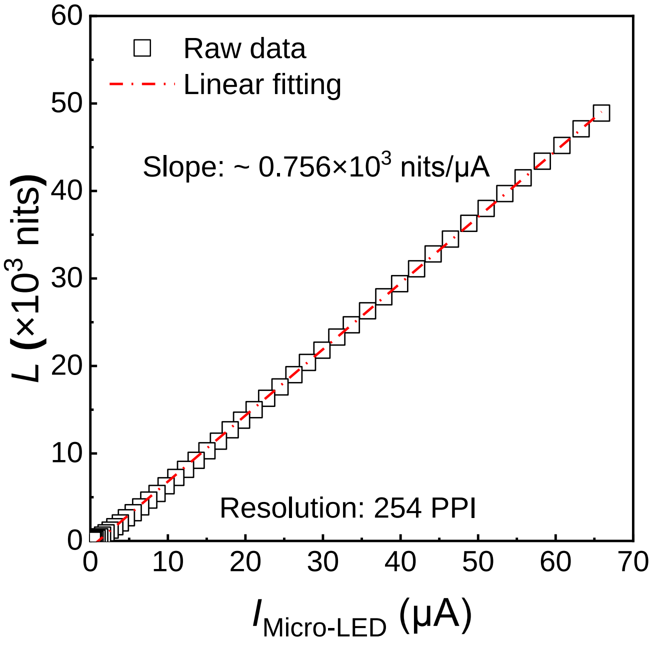

Supplementary Fig. 14 The measured luminance of micro-LED under different driving current for panel with resolution of 254 PPI.

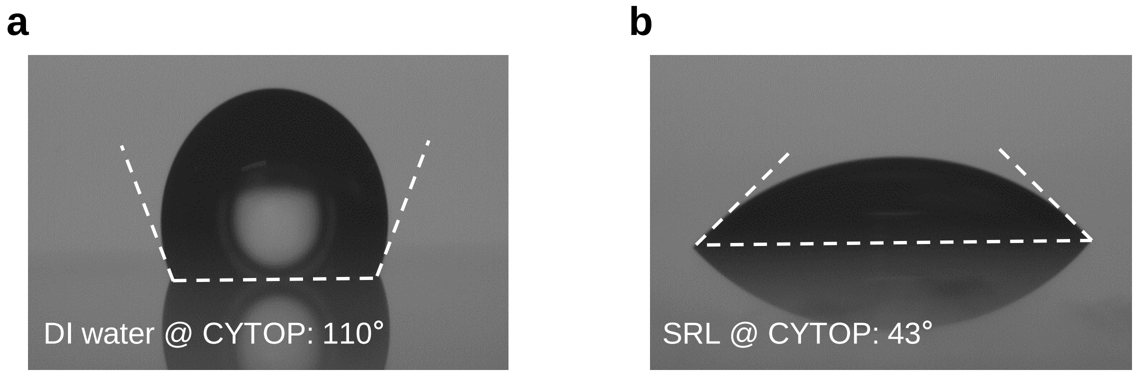

Supplementary Fig. 15 Contact angle characterization: (a) Deionized (DI) water onto hydrophobic CYTOP layer. (b) Sputtering resistant layer (SRL) solution onto hydrophobic CYTOP layer.

Section 2: Supplementary Tables

Supplementary Table 1. Benchmarking of solution-processed OTFT (both OSC and OGI layers) aimed for high current driving capability and high integration density.

Ref.	OSC	OGI	μ ($cm^2V^{-1}s^{-1}$)	L (μm)	μ/L ($\times 10^4$ $cm \cdot V^{-1}s^{-1}$)
1	C_{16}-IDTBT/C_8-BTBT	CYTOP	20.0	100	0.20
2	diF-TES ADT/PTAA	CYTOP	7.2	40	0.18
3	TIPS-Pentacene/PS	PVA	0.8	35	0.02
4	TIPS-Pentacene/PS	SU8	0.4	75	0.005
5	Small molecule (S1200)	Polymer (D207)	1.2	20	0.06
6	Ph-BTNT-C_n	CYTOP	2.2	200	0.01
7	P4FTVT-C32	PMMA	9.4	80	0.12
8	C_{16}-IDTBT	CYTOP	1.4	20	0.07
9	P(NDI2ODT2)	PS	0.7	100	0.007
10	C_8-BTBT/PS	PVP: HDA	43.0	100	0.43
**This work**	**TMTES-Pentacene/Binder**	**CYTOP & SRL**	**2.6**	**3.7**	**0.70**

Supplementary Table 2. Benchmarking of TFT circuit-driven AM micro-LED demonstrations.

Ref.	TFT type	Integration scheme	Highest luminance (cd/m^2)	Process temperature (℃)	Resolution (PPI)
11	IGZO	Mass transfer	6.3×10^2	300	106
12	IGZO	Mass transfer	1.3×10^3	350	42
13	LTPS	Mass transfer	\	450	153
14	LTPS	Mass transfer	9.8×10^2	450	200
15	LTPS	Mass transfer	3×10^3	450	153
16	LTPS	Mass transfer	4×10^3	450	50
17	LTPS	Mass transfer	7×10^2	450	169
18	LTPS	Mass transfer	4×10^4	450	220
19	IGZO	Mass transfer	\	230	70
20	LTPS	Mass transfer	7×10^2	450	228
21	LTPS	Mass transfer	5×10^2	450	72
**This work**	**OTFT**	**Monolithic integration**	**1.5×10^5**	**150**	**254**

*Process temperature of LTPS TFT based on vacuum process is estimated as 450 ℃ according to the standard process in flat panel industry.

Supplementary References

1 Paterson, A., Tsetseris, L., Li, R. et al., Addition of the Lewis Acid Zn(C6F5)2 Enables Organic Transistors with a Maximum Hole Mobility in Excess of 20 cm2V−1s−1. Adv. Mater. 31, 1900871 (2019).

2 Panidi, J., Paterson, A., Khim, D. *et al.*, Remarkable Enhancement of the Hole Mobility in Several Organic Small-Molecules, Polymers, and Small-Molecule:Polymer Blend Transistors by Simple Admixing of the Lewis Acid P-Dopant $B(C_6F_5)_3$. *Adv. Sci.* **5**, 1700290 (2018).

3 Feng, L., Tang, W., Zhao, J. *et al.*, All-Solution-Processed Low-Voltage Organic Thin-Film Transistor Inverter on Plastic Substrate. *IEEE Trans. Electron Devices* **61**, 1175-1180 (2014).

4 Tang, W., Feng, L., Yu, P. *et al.*, Highly Efficient All-Solution-Processed Low-Voltage Organic Transistor with a Micrometer-Thick Low-k Polymer Gate Dielectric Layer. *Adv. Electron. Mater.* **2**, 1500454 (2016).

5 Fukuda, K., Takeda, Y., Mizukami, M. *et al.*, Fully Solution-Processed Flexible Organic Thin Film Transistor Arrays with High Mobility and Exceptional Uniformity. *Sci. Rep.* **4**, 3947 (2014).

6 Kitahara, G., Inoue, S., Higashino, T. *et al.*, Meniscus-Controlled Printing of Single-Crystal Interfaces showing Extremely Sharp Switching Transistor Operation. *Sci. Adv.* **6**, eabc8847 (2020).

7 Bai, J., Jiang, Y., Wang, Z. *et al.*, Bar-Coated Organic Thin-Film Transistors with Reliable Electron Mobility Approaching 10 $cm^2V^{-1}s^{-1}$. *Adv. Electron. Mater.* **6**, 1901002 (2020).

8 Venkateshvaran, D., Nikolka, M., Sadhanala, A. *et al.*, Approaching Disorder-Free Transport in High-Mobility Conjugated Polymers. *Nature* **515**, 384-388 (2014).

9 Li, J., Sun, Z. & Yan, F. Solution Processable Low-Voltage Organic Thin Film Transistors with High-k Relaxor Ferroelectric Polymer as Gate Insulator. *Adv. Mater.* **24**, 88-93 (2012).

10 Yuan, Y., Giri, G., Ayzner, A. *et al.*, Ultra-High Mobility Transparent Organic Thin Film Transistors Grown by an Off-Centre Spin-Coating Method. *Nat. Commun.* **5**, 3005 (2014).

11 Um, J., Jeong, D., Jung, Y. *et al.*, Active-Matrix GaN μ-LED Display Using Oxide Thin-Film Transistor Backplane and Flip Chip LED Bonding. *Adv. Electron. Mater.* **5**, 1800617 (2019).

12 Lin, Y., Liu, C., Zhang, J. *et al.*, Active-Matrix Micro-LED Display Driven by Metal Oxide TFTs Using Digital PWM Method. *IEEE Trans. Electron Devices* **68**, 5656-5661 (2021).

13 Tamaki, M., Yokoyama, R., Aoki, K. *et al.*, LTPS TFT Full Color MicroLED Display with Redundant Pixel Design and Covering Micro-Reflector Array. *SID Symp. Dig. of Tech. Papers* **52**, 29-32 (2021).

14 Nakamitsu, S., Ito, H., Suzuki, T. *et al.*, High PPI Micro LED Display for Small and Medium Size. *SID Symp. Dig. of Tech. Papers* **50**, 137-140 (2019).

15 Tamaki, M., Suzuki, T., Aoki, K. *et al.*, A 3.9-inch LTPS TFT Full Color MicroLED Display with Novel Driving and Reflector Cavity Process. *SID Symp. Dig. of Tech. Papers* **51**, 111-114 (2020).

16 Kim, J., Shin, S., Kang, K. *et al.*, PWM Pixel Circuit with LTPS TFTs for Micro-LED Displays. *SID Symp. Dig. of Tech. Papers* **50**, 192-195 (2019).

17 Sugiura, N., Chuang, C., Hsieh, C. *et al.*, 12.1-inch 169-ppi Full-Color Micro-LED Display Using LTPS-TFT Backplane. *SID Symp. Dig. of Tech. Papers* **50**, 450-453 (2019).

18 Kim, H., Um, J., Lee, S. *et al.*, High Brightness Active Matrix Micro-LEDs with LTPS TFT Backplane. *SID Symp. Dig. of Tech. Papers* **49**, 880-883 (2018).

19 Yang, J., Park, H., Kim, B. *et al.*, Active-Matrix Micro-Light-Emitting Diode Displays Driven by Monolithically Integrated Dual-Gate Oxide Thin-Film Transistors. *J. Mater. Chem. C* **10**, 9699-9706 (2022).

20 Kim, H., Um, J., Lee S. *et al.*, High Brightness Active Matrix Micro-LEDs with LTPS TFT Backplane. *SID Symp. Dig. of Tech. Papers* 49, 880-883 (2018).

21 Jung, H., Park, C., Gee, M. *et al.*, Active Matrix Micro-LED Stretchable Display and Technical Challenges. *SID Symp. Dig. of Tech. Papers* **53**, 517-520 (2022).
