# Supplementary material for: Wafer-scale organic-on-III-V monolithic heterogeneous integration for active-matrix micro-LED displays
Source: Nat Commun. 2023 Nov 1;14:6985. doi: 10.1038/s41467-023-42443-8 (PMC10620182; doi:10.1038/s41467-023-42443-8)
Supplement: Supplementary file 3 — Description of Additional Supplementary Files [file 41467_2023_42443_MOESM3_ESM.pdf]

File name: Supplementary Movie 1

Description: OTFT based AM microLED display with resolutions of 254 PPI.
